# The effect on knee-joint load of instruction in analgesic use compared with neuromuscular exercise in patients with knee osteoarthritis: study protocol for a randomized, single-blind, controlled trial (the EXERPHARMA trial)

**DOI:** 10.1186/1745-6215-15-444

**Published:** 2014-11-15

**Authors:** Brian Clausen, Anders Holsgaard-Larsen, Jens Søndergaard, Robin Christensen, Thomas P Andriacchi, Ewa M Roos

**Affiliations:** Research Unit for Musculoskeletal Function and Physiotherapy, Institute of Sports Science and Clinical Biomechanics, University of Southern Denmark, Odense, Denmark; Orthopaedic Research Unit, Department of Orthopaedics and Traumatology, Odense University Hospital, Institute of Clinical Research, University of Southern Denmark, Odense, Denmark; Research Unit for General Practice, Institute of Public Health, University of Southern Denmark, Odense, Denmark; Musculoskeletal Statistics Unit (MSU), The Parker Institute, Department of Rheumatology, Copenhagen University Hospital, Bispebjerg and Frederiksberg, Copenhagen, Denmark; Departments of Mechanical Engineering and Orthopaedic Surgery, Stanford University, Stanford, California USA; VA Joint Preservation Center, Palo Alto, California USA

**Keywords:** Osteoarthritis, Exercise, Gait, Joint load, Knee joint, Middle-aged, Knee, Pain management

## Abstract

**Background:**

Knee osteoarthritis (OA) is a mechanically driven disease, and it is suggested that medial tibiofemoral knee-joint load increases with pharmacologic pain relief, indicating that pharmacologic pain relief may be positively associated with disease progression. Treatment modalities that can both relieve pain and reduce knee-joint load would be preferable. The knee-joint load is influenced by functional alignment of the trunk, pelvis, and lower-limb segments with respect to the knee, as well as the ground-reaction force generated during movement. Neuromuscular exercise can influence knee load and decrease knee pain. It includes exercises to improve balance, muscle activation, functional alignment, and functional knee stability. The primary objective of this randomized controlled trial (RCT) is to investigate the efficacy of a NEuroMuscular EXercise (NEMEX) therapy program, compared with optimized analgesics and antiinflammatory drug use, on the measures of knee-joint load in people with mild to moderate medial tibiofemoral knee osteoarthritis.

**Method/Design:**

One hundred men and women with mild to moderate medial knee osteoarthritis will be recruited from general medical practices and randomly allocated (1:1) to one of two 8-week treatments, either (a) NEMEX therapy twice a week or (b) information on the recommended use of analgesics and antiinflammatory drugs (acetaminophen and oral NSAIDs) via a pamphlet and video materials. The primary outcome is change in knee load during walking (the Knee Index, a composite score of the first external peak total reaction moment on the knee joint from all three planes based on 3D movement analysis) after 8 weeks of intervention. Secondary outcomes include changes in the external peak knee-adduction moment and impulse and functional performance measures, in addition to changes in self-reported pain, function, health status, and quality of life.

**Discussion:**

These findings will help determine whether 8 weeks of neuromuscular exercise is superior to optimized use of analgesics and antiinflammatory drugs regarding knee-joint load, pain and physical function in people with mild to moderate knee osteoarthritis.

**Trial registration:**

ClinicalTrials.gov Identifier: NCT01638962 (July 3, 2012).

## Background

Knee osteoarthritis (OA) is a common chronic joint disease leading to pain and loss of physical function, resulting in reduced quality of life [1]. About one third of people with knee OA will experience progression to more-advanced disease [2], which is the leading indication for knee-replacement surgery. In western countries, the Age-Standardized Incidence Rates for total knee replacement are crudely 150 per 100,000 person-years [3]. However, the majority of patients do not progress and are managed in primary health care.

Clinical guidelines advocate nondrug treatments as first-line treatment for knee OA [4–6]. These include information, exercise, and weight loss, and are preferred for their anticipated negligible adverse effects while still having relevant clinical efficacy. Despite this, both over-the-counter and prescribed pain-reducing pharmacologic agents (analgesics and antiinflammatory agents) are widely and more commonly used treatments for knee OA in primary health care [7]. Although these are preferred for their ease of application and dose-dependent pain-relieving effect [8], they also have dose-dependent adverse effects [9–11].

### Biomechanical factors play a role in symptoms and disease progression in knee OA

After the use of analgesic agents, pharmacologic pain relief seems to be associated with increased joint loads, which can potentially result in disease progression [12, 13]. Joint loading has a central role in symptoms and disease progression [14], and therapies targeting mechanical load are thus likely to be successful in the management of knee OA. During the stance phase of walking, high loads are applied to the medial knee compartment [15]. Some evidence shows that the external peak knee-adduction moment (KAM) has a significant relation to *in vivo* measurement of medial compartment load [15], and important clinical outcomes, including radiographic OA severity [16] and knee pain [17]. One study showed that the KAM impulse might be more sensitive in discriminating between the OA severity level than the peak KAM [18]. A novel study showed that the combined peak knee moment from all three planes (Knee Index) is sensitive enough to distinguish between pain levels [19]. With pharmacologic and biomechanical treatments, the joint load is amenable to clinically relevant change [19–23].

The knee index is influenced by the functional alignment of the trunk, pelvis, and lower-limb segments with respect to the knee during movement and the ground-reaction force generated. Thus, it is likely that interventions, such as neuromuscular exercise, targeting the efficiency of lower-limb movement and muscle-activation patterns can be effective in improving dynamic knee-joint loading [14, 24], whereas interventions providing pain relief through pharmacologically mediated pathways may be associated with an increase in knee-joint load [12, 13].

### Exercise as treatment in knee injury and knee OA

NEuroMuscular EXercise (NEMEX) includes exercises to improve balance, muscle activation, functional alignment, and functional joint stability. Unlike conventional strength training, neuromuscular exercise addresses the quality of movement and emphasizes joint control in all three planes. It has effects on knee functional performance, knee biomechanics, and muscle-activation patterns of the surrounding knee musculature [25, 26]. Neuromuscular exercise increases functional knee stability and, in pilot studies, has shown potential to reduce knee-joint loads and improve cartilage matrix quality in those at risk or with mild disease [27–29] but not in those with malaligned knees and more advanced disease [30]. Neuromuscular exercise is used effectively for prevention and rehabilitation of anterior cruciate ligament injury [31–33] rehabilitation in patients with a meniscal tear with or without the combination of meniscectomy [34, 35] and in patients with moderate to severe OA before total joint replacement [36, 37]. Despite its use in other conditions and in more-severe stages of OA, only one study has investigated the effect of this exercise form in early stages of knee OA: an uncontrolled pilot study consisting of 13 patients with mild knee OA [27] that resulted in a −0.8 Nm/kg (95% CI, −0.04 to -0.16) reduction (14%) in peak KAM during one-leg rise after 8 weeks of neuromuscular exercise.

### Pharmacologic pain relief and joint load in knee OA

Pain is a mechanism that helps to protect damaged tissue and tissue at risk of damage. In healthy subjects, experimentally induced knee pain has been shown to replicate altered gait-movement strategies seen in patients with mild knee OA (that is, reduced first peak KAM and sagittal plane moments were seen) [38]. Studies have shown that pharmacologically initiated pain relief in knee OA is associated with increased loads in KAM and sagittal knee moments [12, 17, 32, 39, 40]. Therefore, pharmacologic pain relief, by eliminating the protective mechanism of the pain itself may be detrimental for knee-joint structures by increasing knee-joint load. The most common pharmacologic pain relief used in the management of knee OA is acetaminophen and nonsteroidal antiinflammatory drugs (NSAIDs). The pain-relieving effect seen from acetaminophen and NSAIDs is comparable with the pain-relieving effect from a low dose (less than 12 sessions) of exercise [41, 42].

### Objectives

The primary objective of this study is to compare the efficacy of a specific neuromuscular exercise program with optimized analgesics and antiinflammatory drug use on knee loads, as well as pain and physical function in people with mild to moderate medial tibiofemoral knee OA.

### Primary hypothesis

The first peak Knee Index during walking will be reduced more by neuromuscular exercise than by analgesic use.

### Secondary hypotheses

Additional measures of knee-joint loading (Knee Index, Knee Adduction Moment (KAM) and KAM impulse) during one-leg stands from a stool and/or during walking will be reduced more by neuromuscular exercise compared with analgesic use. Functional performance will be significantly improved by neuromuscular exercise in comparison with analgesic use. Pain relief will be equivalent or potentially superior in the group having had neuromuscular exercise.

## Methods/Design

### Trial design and setting

**Figure 1:**
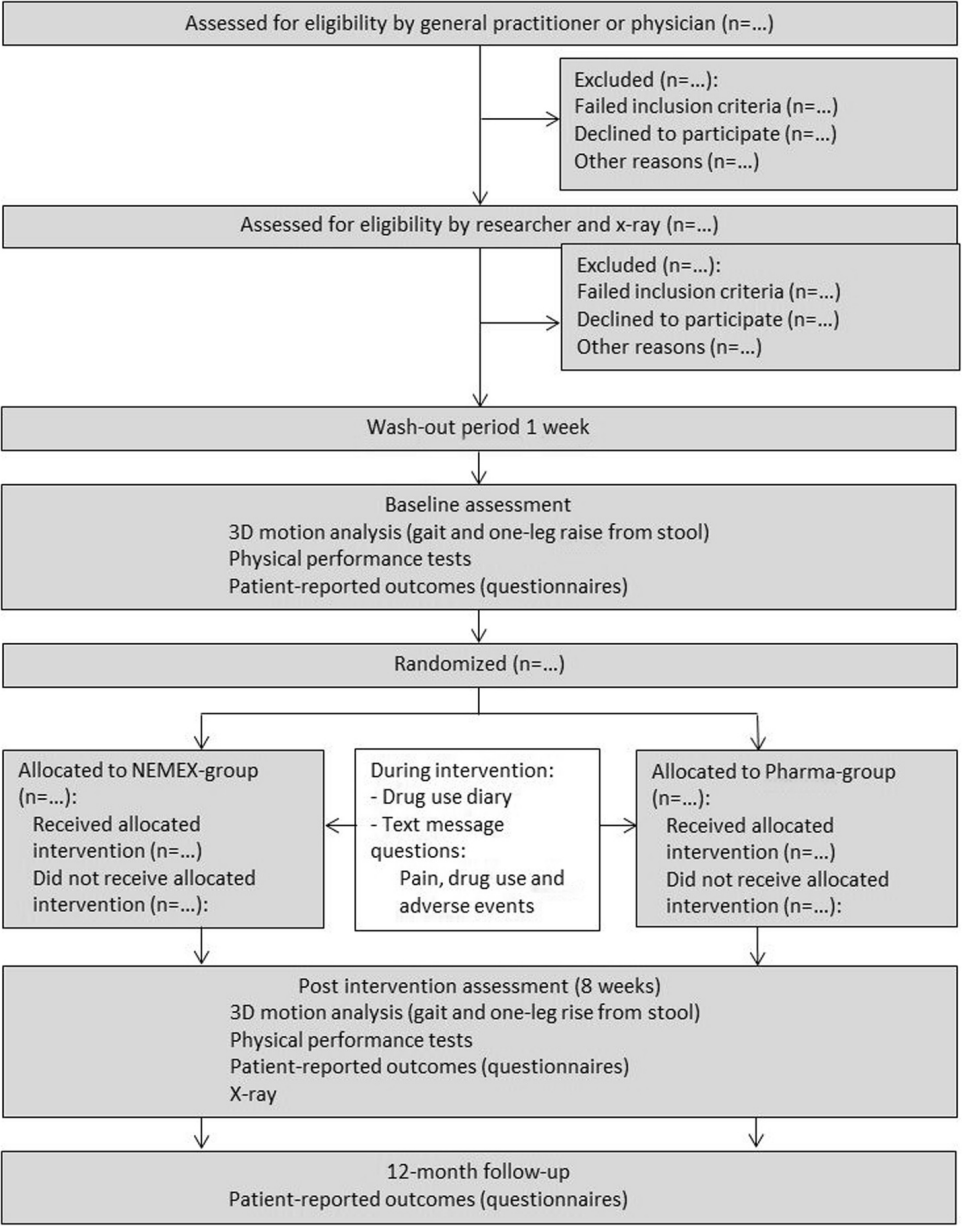
**Flow diagram.**

This is a single-center, unstratified (with balanced randomization (1:1)), single‒blind, controlled, parallel‒group study conducted in Denmark. The study protocol conforms to the SPIRIT statement [43], and the subsequent reporting will follow the recommendations from CONSORT for nonpharmacologic studies [44] (Figure 1: Flow diagram). Participants will be recruited from general medical practices [45] and advertisements in the local community.

### Participants

A sample of 100 patients with knee OA (including both men and women) aged 40 to 70 years will be recruited via primary care general practitioners (GPs) in the communities of Odense and Middelfart, Denmark, and from advertisements in local clubs, libraries, print media, and Facebook. For a full list of inclusion and exclusion criteria, see Table 1. In summary, the eligibility criteria are selected to achieve high external validity of the study findings by using a pragmatic trial design [46]. Patients should have persistent knee pain in accordance with the ACR criteria [47] and no contraindication for exercise, NSAIDs, or x-ray, or have had leg surgery/trauma within the last 6 months.

**Table 1:** **Eligibility criteria of the EXERPHARMA-trial**

**Inclusion criteria**	
1.	Compliance with the ACR criteria [47, 48]
a)	Risk factors: Age >40 years; female; overweight; occupation; family history of OA
b)	Symptoms: persistent knee pain; brief morning stiffness; functional limitations; acute knee pain
c)	Objective examination: crepitus during active movement; bony tenderness; bony enlargement; palpable effusion; no palpable warmth; restricted movement; instability.
2.	No, mild, or moderate medial knee OA defined as “No osteoarthritis”, “Doubtful narrowing of joint space and/or possible osteophytes”, “Definite osteophytes and possible narrowing of joint space”, “Multiple osteophytes, definite narrowing of joint space, and some sclerosis and deformity of bone ends.” This corresponds to the modification of Kellgren and Lawrence (KL) grades 0, 1, 2, and 3, respectively [49, 50]
3.	Willingness to participate in exercise intervention and pharmacologic intervention
4.	A maximum of 80 of 100 points in the KOOS Pain subscale (corresponding to, on average, at least mild pain)
5.	BMI of less than 32
**Exclusion criteria**	
General:	
1.	Difficulty complying with treatment schedule
2.	Inability to fill out questionnaires
3.	Inability to ambulate without assistive device
4.	Problems affecting the lower extremity overriding the problems from the knee
5.	Physician-determined:
6.	Any condition contraindicating use of acetaminophen, NSAIDs, or exercise
7.	Already taking NSAIDs or acetaminophen at doses similar to or higher than the study dose
8.	Diagnosis of systemic arthritis
Radiographic:	
1.	Medial greater than lateral joint-space width
2.	Medial knee OA of KL grade 4
Previous and planned interventions:	
1.	Previous ACL reconstruction or known ACL deficiency
2.	Previous tibial osteotomy
3.	Previous ankle, knee, or hip total joint replacement
4.	Knee surgery including arthroscopy within the past 6 months
5.	Steroid injection within the past 6 months
6.	Knee surgery planned within the next 6 months

### Procedure, randomization and allocation concealment, and blinding

Participants will be given a short introduction to the study and be assessed for eligibility by a GP. The GPs will be recruited by a letter of invitation and given an honorarium (€35) for each included participant. Participants recruited through advertisements will be assessed for eligibility by a physiotherapist. Thereafter, the participant will be invited to a formal information meeting with the project manager, during which the signing of an informed-consent form, and a clinical assessment will take place for all participants.

Eligible participants will be randomly allocated in permuted blocks of four to six generated *a priori* by our statistician (RC) to either the group receiving the NEMEX therapy (NEMEX-group) or the group receiving the analgesics and NSAIDs therapy (Pharma-group). See later for description of the two interventions. Consecutively numbered opaque envelopes will be opened after the participant has been tested at baseline (BC). The patient will be informed about the allocation immediately after baseline testing.

All outcomes will be assessed at baseline and after treatment (8 weeks), and the self-reported measurements will also be collected by mail at 12 months (see Figure 1). Outcome assessments at baseline and after treatment will be carried out at the motion laboratory at the Odense University Hospital by the same assessors who will remain blinded to group allocation throughout the study. Data analysis will be done in a blinded manner by the study statistician (RC) not directly involved in the study.

### Interventions

#### The NEMEX-KOA (NEuroMuscular Exercise–Knee Osteo Arthritis) training program

**Figure 2:**
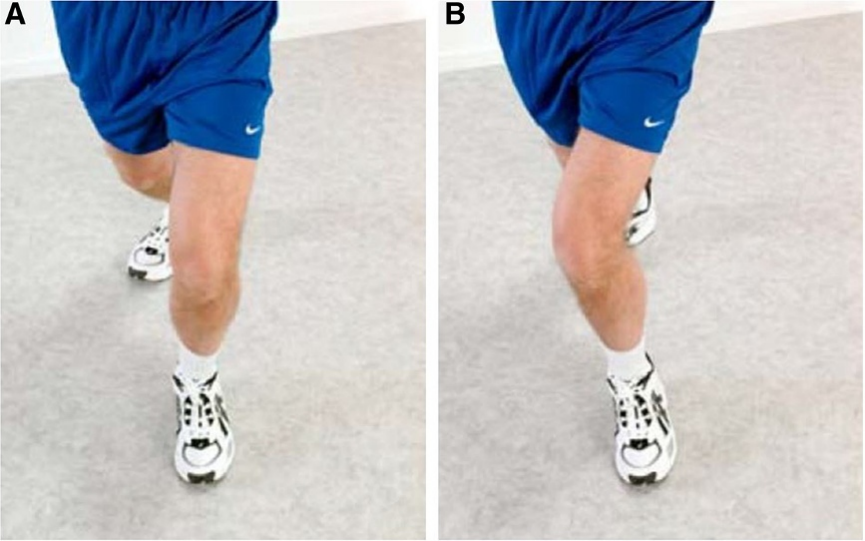
**Knee alignment. (A)** “Knee-over-toe-position”, that is, lower extremity well aligned with appropriate position of knee over foot. (**B)** “Knee-medial to-foot-position”, that is, lower extremity not well aligned; the medial placement of the knee relative to the foot is inappropriate.

We have applied the principles of neuromuscular training in the NEMEX-KOA training program as follows: The training sessions consist of five parts: warming up, functional, proprioceptive, endurance strengthening, and cooling down. The warming-up period consists of ergometer cycling, treadmill, or stepper for 10 minutes at a ”rather strenuous” level [51]. The functional part comprises five exercises, including neuromuscular exercises with the key elements: core stability/postural function, postural orientation, lower-extremity muscle strength. *The* proprioceptive part comprises three exercises, with the key elements being balance and functional stability (Figure 2). The endurance strengthening part comprises three exercise circuits, with the key elements of postural and functional stability of the trunk and knee. Some of the constructs related to muscle function, muscle tests, and muscle training (adapted from Ericsson et al. [35]) are outlined in Table 2.

**Table 2:** Constructs related to muscle function

Constructs	Definitions
Muscle strength	The amount of external force that a muscle can exert
Muscular endurance	The ability of muscle groups to exert external force for many repetitions or successive exertions
Functional alignment	Lower limb alignment during weight-bearing. A correct functional alignment means that the knee is lined up over the second toe without tending to fall inwards/medially during knee flexion (Figure 2)
Functional performance test/measures	A test that challenges muscle strength and postural control or dynamic joint stabilization in the lower extremities and the trunk (for example, various knee bending tests). A physical performance test is a quantitative test (for example, measuring length, number of repetitions, seconds) that evaluates the prerequisite for function
Closed kinetic chain exercise	Weight-bearing exercise with distally situated axis of motion and a movement occurring in several joints, and where the distal segment is usually fixed to a supporting surface (for example, a squat)
Open kinetic chain exercise	Non weight-bearing exercise, with a proximally situated axis of motion and a movement occurring at a single joint, and where the distal segment is free to move (e.g., extension of the knee in a sitting position)

The exercises are mainly performed in a closed kinetic chain. However, because muscle weakness of the lower extremity, particularly the quadriceps, is common in participants with OA, open kinetic chain exercises are also used to improve muscle strength of the knee, hip and trunk muscles. Each exercise is performed in two sets of 12 repetitions, with the time for rest corresponding to the duration of one set, between sets. The exercises are performed with both the non-affected and the affected leg, although focus is on the affected leg. To allow for progression, three to four levels of difficulty are given for each exercise. Progression is made when an exercise is performed with good sensorimotor control and good quality of performance (based on visual inspection by the physiotherapist) and with minimal exertion and control of the movement (perceived by the participant). The *cooling down* part of the training program includes walking in various ways with emphasis on alignment, and stretching exercises for the lower extremity muscles (10 minutes) [34, 52, 53].

The exercises in the five parts of the training program are given in File 1 of the appendix. Training will take place in groups, at one of two clinics under the supervision of one of two experienced physiotherapists specialized in the treatment of musculoskeletal disorders. All treating physiotherapists in this study have received education in the exercise program and the study’s data-registration process; in addition, they are supervised by a colleague and have regular meetings with the first author (BC) to ensure compliance with the study protocol and the exercise program. On average, about 5 to 10 participants will attend each training session. New participants will continuously enter the training group that is, the group will hold both novice participants and those who have participated in a number of the training sessions and, thus, will be more familiar with the training. During each group-training session, each participant will be monitored individually so that the exercises are performed at a training level consistent with their current level of neuromuscular function.

The participants will be offered two supervised training sessions per week, each of 60 minutes. The training sessions will take place in the late afternoon, because half of the participants are anticipated to be active members of the work force. Based on earlier studies, the intervention period will be 8 weeks, with sessions run twice per week (up to a maximum of 16 sessions) [52, 54].

### Pain monitoring during the exercise intervention

The pain-monitoring system is part of the NEMEX concept [55]. Participants will report pain on a visual analogue scale (VAS) graded from 0 to 10, in which 0 is “no pain” and 10 is “pain as bad it could be”, before and after each training session. Pain up to 2 on the scale is considered “safe”; between 2 and 5 is considered “acceptable”; and pain >5 is considered “high risk” [52, 55]. “Acceptable pain” (between 2 and 5) is allowed during and immediately after training, and increase in resting pain compared with normal is accepted as long as the increase has subsided to normal resting pain level at 24 hours after the training session [22, 52, 55].

### Compliance with the exercise therapy program

All possible effort will be made to enhance compliance with the program, such as explaining the importance of adhering to the exercises to receive effect.

### Registration of adherence, exertion, and pain in the NEMEX group

At every exercise session, the physiotherapist will record, in an exercise diary, the date for each attended session (to determine the number of sessions), the individual level for each exercise (to determine progression from level to level), perceived exertion (Borg RPE CR-10 [51]), and current pain (VAS). If participants perceive unacceptable pain during or after exercise, the supervising physiotherapist will assist in decreasing the level of the exercises.

### Rescue medication in the NEMEX group

Although we do not recommend it, participants are allowed over-the-counter and prescribed pharmacologic pain relief as rescue medication. Use of rescue medicine will be noted by the participants in their individual diaries.

### Pharmacologic pain-relief (Pharma) group

Participants in the Pharma group will receive information on how best to use acetaminophen and oral NSAIDs, in doses consistent with Danish guidelines [4]. The information will be provided by a pamphlet and a video outlining the recommended use of mild analgesics and antiinflammatory drugs (that is, acetaminophen and oral NSAIDs). Osteoarthritis Research Society International (OARSI), European League Against Rheumatism (EULAR), and Danish guidelines recommend starting treatment with acetaminophen up to 4 g/daily in three to four doses. If acetaminophen proves to be inadequate, the treatment can be supplemented with an oral NSAID [4–6]. For participants with an increased risk of gastrointestinal problems, a mucosal protector will be recommended in addition to the NSAID. Participants will be encouraged to take their medication according to need and can change the medication when their pain levels alter.

Treatment in the Pharma group is designed to reflect recommended use of acetaminophen and over-the-counter NSAIDs. Therefore, participants will have to pay for their own drugs. In Denmark, where the trial will take place, the cost for full-dose (4,000 mg daily for 8 weeks) use of acetaminophen will be €30 (at 2013 prices), and for full-dose (2,400 mg daily for 6 weeks) NSAIDs (for example, Ibuprofen), the cost will be €30. If participants do not have sufficient pain relief from over-the-counter acetaminophen, the information pamphlet will inform them to contact their GPs, who may prescribe additional NSAIDs. The GP has been instructed that the Pharma group in this study is to use either acetaminophen alone or acetaminophen in combination with NSAIDs.

### Monitoring during the treatment period

Both groups will be contacted by Short Message Service (text messaging) on their mobile phones twice per week (in total, 16 assessments) and will be asked to answer three questions: 1. “What is your level of pain right now?” (0 = none, 1 = mild, 2 = moderate, 3 = severe and 4 = extreme); 2. “Did you use any pain-relieving drug yesterday?” (yes/no); 3. “Have you had any adverse event since the last text message?” (yes/no), and if yes to Question 3, the participant will be contacted by the first author (BC) to identify the nature of the adverse event. With this monitoring, we will be able to know how pain changes during the treatment period, how often analgesic drugs are used during the treatment period, and if and when adverse events occur during the treatment period. Text messaging has been successfully used previously to evaluate real-time back pain [56].

### Outcome measures

Outcome measures are listed in Table 3.

**Table 3:** **Outcome measures in the EXERPHARMA trial**

	Collection time points
**Primary end point**	
Knee biomechanics (mean ±95% CI):	0, 8 weeks
Knee index, during gait	
**Secondary end points:**	
Knee biomechanics (mean ±95% CI):	0, 8 weeks
Knee Index, during one-leg rise	
Peak Knee Adduction Moment, during gait	
Peak Knee Adduction Moment, during one-leg rise	
Knee Adduction Moment Impulse, during gait	
Knee Adduction Moment Impulse, during one-leg rise	
Functional performance test (mean ±95% CI):	0, 8 weeks
Maximum one-leg rises from stool	
Maximum number of knee-bendings in 30-second test	
One-leg hop for distance test	
Patient-reported outcomes:	
Mean KOOS subscale scores (mean ±95% CI):	0, 8, 52 weeks
Pain	
Other symptoms	
Activities of Daily Living (ADLs)	
Sport and Recreation Function	
Knee-related Quality of Life (QOL)	
Activity level:	
UCLA activity score, change from baseline	0, 8, 52 weeks
Pain level:	
Pain-level text messages, intensity (0–4)	During treatment

#### Primary outcome

The primary outcome is change in knee load during walking. In this article, the term knee load refers to first peak Knee Index, a surrogate measure calculated to estimate the knee load. The primary outcome will be between-group change in Knee Index immediately after intervention.

**Figure 3:**
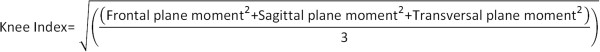
**Equation for calculating the Knee Index.**

*External Peak joint moments* in the frontal, sagittal, and transverse plane will be used to calculate the Knee Index during gait. The Knee Index is a surrogate for total load across both compartments, and has been chosen because changes in the external moment are known to occur in the sagittal plane before and without accompanying changes in the frontal and/or transverse plane [14, 19, 57]. Knee index is a novel functional variable, constructed from the maximal external moments affecting the knee in the frontal, sagittal, and transverse planes, expressed in Figure 3 [58]. The Knee Index will be reported normalized to height and weight.

### Secondary outcomes

#### Biomechanical outcomes

The secondary biomechanical outcomes will be the Knee Index during one-leg rise, first peak Knee Adduction Moment (KAM) during gait and one-leg rise, and KAM impulse during gait and one-leg rise. These measures will be normalized to height and weight.

All biomechanical outcome calculations will be based on measurements taken during gait and one-leg rise before and after treatment by using a 3D Vicon MX movement-analysis system with eight cameras operating at 100 Hz (Vicon, Oxford, UK) and two AMTI force-plates (AMTI, OR6-7, Watertown, MA, USA) embedded at floor level, operating at 1,000 Hz. Because of hardware upgrade during the inclusion period, the first 24 subjects will be measured with a 3D Vicon MX movement-analysis system with six cameras operating at 100 Hz (Vicon) at both baseline and after intervention. A technician experienced in gait analysis and the Vicon system will attach reflective markers that reflect infrared light according to the Vicon Plug-in-Gait marker set and model [59, 60]. Data will be combined by using inverse dynamics to yield measures of external joint moments and ranges of motion and calculated by Plug-In Gait software.

### Functional performance measures

To avoid systematic bias due to a potential learning effect, the leg to be tested first will be randomized at the time of each testing.

To evaluate functional performance [61], we will use three tests that have shown evidence of validity in knee OA, in the following order; Maximum number of one-leg rises from a stool [62], maximum number of knee-bendings in 30 seconds [63], and one-leg hop for distance [63].

### Maximum one-leg rises from a stool

This test evaluates the number of times the participant can rise from a stool on one leg [27, 62, 64]. A lower number of one-leg rises has been found to be predictive of development of radiographic signs of OA [62]. The participant sits on a stool (0.48 m). The participant places one foot on the floor in a self-chosen position. One familiarization attempt is allowed, and thereafter, the foot position may not be changed during the test. The lifted leg is held with straight knee, and the arms hang alongside the body. The test is to be performed with full muscle control—that is, the sitting-down phase should be performed at constant speed and the rising phase is to be performed without adding arm or trunk movement. A pause of 5 minutes is allowed between testing the right and left leg. If the opposite (raised) leg touches the ground, the trial is stopped, and the performed number is noted. The number of adequately performed rises is counted and noted [27, 62]. This test will also be recorded with 3D motion capture and video from the right side.

### Maximum number of knee-bendings in 30 seconds

This test evaluates the ability to perform fast changes between eccentric and concentric muscle force over the knee joint, and is found to be reduced in participants with and without radiographic OA at 20 years after meniscectomy compared with healthy controls [63, 65]. The participant stands on one leg with fingertip support of the index fingers from a bar placed in front, with the foot placed on the vertical line of a T-mark on the floor. The participant then looks down on the horizontal line and bends his or her knee, without bending forward from the hip, until he or she no longer can see the horizontal line (about 30 degrees of knee flexion) consecutively for 30 seconds [63]. The participant will first perform practice trials for 10 seconds. Thereafter, the stopwatch will be started after a countdown from three. For each leg, the total number of knee bends performed in 30 seconds is noted. If the opposite (raised) leg touches the ground, the trial is stopped, and the performed number is noted [63]. This test will also be recorded with 3D motion capture and video from the front.

### One-leg hop for distance

This test mimics sporting activities and demands explosive muscle function, balance, and functional stability of the knee, and was found feasible in participants with a mean age of 50.3 years with and without radiographic OA at 20 years after meniscectomy [63, 66, 67]. The participant stands on the leg to be tested, hops, and lands on the same limb. The hands are placed behind the back. The participant is instructed to perform a controlled, balanced landing and to keep the landing foot in place for 2 to 3 seconds until the landing position has been recorded by the tester. Failure to stand still for 2 to 3 seconds results in a disqualified hop. The distance hopped is measured in centimeters from the toe at push-off to the heel where the participant lands. Participants will perform one practice trial and then three test trials. If the participant improves more than 10 cm between the second and third hops, an additional hop is performed [63, 66]. Shoes will be worn. The best trial will be used. Symmetry index will be calculated as (injured side/uninjured side) × 100 [66].

### Patient-reported outcomes

The Knee injury and Osteoarthritis Outcome Score (KOOS, http://www.koos.nu) [68–70] is a questionnaire that assesses self-reported outcomes in five separate subscales: Pain, Other symptoms, Activities of Daily Living (ADL), Sport and Recreation Function, and Knee-related Quality of Life (QOL), calculated as separate subscale scores ranging from 0 to 100, worst to best. For assessment of physical-activity level, the University of California at Los Angeles (UCLA) activity score will be used [71]. It assesses self-reported current activity level on a scale of 1 to 10, worst to best.

### Exploratory outcome measures

Exploratory outcome measures are listed in Table 4.

**Table 4:** **Other descriptive data collected in the EXERPHARMA trial**

	Collection time points
**Descriptive data**	
Aastrands test	0, 8 weeks
Observer-reported outcomes	
Performance in exercise group	
Exercise diary (progression, pain, exertion level)	During treatment
Patient-reported outcomes	
Generic health measure	
SF-36 acute v. 1.0 (95% CI)	0, 8, 52 weeks
Health economic evaluation	
EQ-5D v. 1.0 (95% CI)	0, 8, 52 weeks
Adverse events	
Adverse events questionnaire, change from baseline	0, 8 weeks
Adverse events text messages, number and types of incidents	During treatment
Drug use	
Drug use diary, amount, intensity, and type	8 weeks
Drug use text message, numbers	During treatment
Assessment of treatment	
Global perceived effect (GPE)	8, 52 weeks
Patient Acceptability Symptom State (PASS)	8, 52 weeks
Treatment since end of study treatment	
Treatment questionnaire, amount, type, and duration	52 weeks

#### Aerobic capacity

##### Aastrands test

Before the 3D movement analysis and functional performance tests, participants will perform a standardized warm-up session consisting of the Aastrands test (submaximal 1-point test on a stationary bicycle) for approximately 10 minutes. The participant cycles at 60 rpm, with the workload adjusted to target a stable heart rate of 120 to 170 bpm. From pulse rate and workload, the aerobic capacity will be estimated [51].

### Radiographic outcomes

#### Classification of osteoarthritis

As a means of describing participant characteristics, the radiographic severity will be classified according to the Kellgren-Lawrence (KL) classification in grades 0 to 3, with grade 4 being an exclusion criterion (Table 5) [49, 50]. Radiographs of both knees will be taken, in posterior-anterior, mediolateral directions, and patella skyline. The posterior-anterior radiograph is taken with the participant standing at a fixed flexion angle by using the Synaflex frame [72]. The mediolateral radiograph is taken with the participant standing with semiflexed knees (~10 degrees) and the tibia kept vertical [73]. The patella skyline radiograph is taken with the participant standing with flexed knee (about 70 to 110 degrees), with the x-ray beam level with the base of the patella [73].

**Table 5:** **The Kellgren-Lawrence classification for osteoarthritis**

Grade of osteoarthritis	Description
0	No osteoarthritis
1	Doubtful narrowing of joint space and/or possible osteophytes
2	Definite osteophytes and possible narrowing of joint space
3	Multiple osteophytes, definite narrowing of joint space, and some sclerosis and deformity of bone ends
4	Large osteophytes, marked narrowing of joint space, severe sclerosis, and definite deformity of bone ends

### Additional patient characteristics

At screening, the participants will fill out a questionnaire to record gender, age, weight, height, level of education, and working, smoking, and civil status.

### Health economic analyses

To perform a health economic evaluation, the participants will complete the EuroQOL (EQ-5D). EQ-5D [74] is a utility index measuring the five dimensions of anxiety, mobility, self-care, pain, and usual activities. The five-domain questionnaire classifies 243 different health states by using three levels of severity for each domain (no problems, some problems, extreme problems). The Danish EQ-5D tariff was estimated by using the time trade-off method in a sample of 1,332 respondents from the Danish general population [75]. It consists of a set of numbers that indicates the level of health-related quality of life for each EQ-5D health state, on a scale from 1 (full health) to 0 [dead; range, −0.624 to 1, where negative values are valued as worse than dead] [75].

### General health

The short-form health survey (SF-36) [76] is a generic measure of health status measuring physical and mental aspects in eight different subscales, calculated in separate subscale scores ranging from 0 to 100, worst to best.

### Adverse events

Reporting of adverse events will be elicited with a nonleading questionnaire at baseline and after treatment. All events will be coded according to the Medical Dictionary for Regulatory Activities, as currently required by all regulatory authorities, including the US Food and Drug Administration and the European Agency for the Evaluation of Medicinal Products [77]. In addition to the questionnaire after treatment, as previously described, adverse events during treatment will be monitored by text messages.

### Drug use

In both treatment groups, participants will complete a drug-use diary, in which they will be asked to fill in date, time, type, and amount of any given drug they have used every day during the treatment period. In addition, they can fill in the reason for taking the drug (for example, headache or knee pain).

### Treatment since end of study treatment

At the 1-year follow-up, the participants will be asked to fill out a questionnaire on what other treatments they may have received since the end of the study treatment.

### Global rating scales

Five Global Perceived Effect (GPE) questions are used for measuring the participants’ overall experience with the intervention. One anchor question is used for determining Patient Acceptable Symptom State (PASS; satisfaction with the current situation) [78]. The five GPE questions are as follows.

#### Pain

How do you experience your knee pain now, compared with 2 months ago when you entered the study? Answer categories: Much less, less, the same, worse, much worse.

#### Symptoms

How do you experience your other knee symptoms now, compared with 2 months ago when you entered the study? (swelling, stiffness, decreased range of motion) Answer categories: Much less, less, the same, worse, much worse.

#### ADL

How is your ability to perform activities of daily living now, compared with 2 months ago when you entered the study? (sitting, standing, walking, ascending/descending stairs, putting on clothes, household activities) Answer categories: Much better, better, the same, worse, much worse.

#### Sport/Recreation

How is your ability to perform sport and recreational activities now, compared with 2 months ago when you entered the study? (running, jumping, squatting, kneeling, twisting/pivoting on injured knee) Answer categories: Much better, better, the same, worse, much worse.

#### QOL

How much do your knee problems affect your quality of life now, compared with 2 months ago when you entered the study? (trust in your knee, changed lifestyle, how often you think of your knee, and so on) Answer categories: Much less, less, the same, worse, much worse.

The anchor question for determining PASS is as follows:

When you take into consideration your daily life, your pain and other symptoms, and your impairment and quality of life, do you then consider your current situation satisfactory? Answer categories: yes, no.

### Sample size

For a two-sample pooled *t* test of a normal mean difference with a two-sided significance level of 0.05, assuming a common standard deviation of 0.8 Nm/BW × HT% [19], a sample size of 42 participants per group is required to obtain a power of at least 80% to detect a difference between the means of first peak Knee Index of 0.5 Nm/BW × HT% (corresponding to a 27% difference in means) [19]. To make up for some attrition during the trial period, we have decided to include 100 participants in total (randomized 1:1). If the drop-out rate proves to be lower, the number of recruited participants will be adjusted accordingly but will not be below 84.

### Data and statistical analysis

Data will be analyzed in a blinded manner. Main comparative analyses between groups will be performed by using a modified intention-to-treat analysis (all cases with available baseline data carried forward). Between-group mean differences and 95% confidence intervals will be estimated with a general linear model in which the participant’s baseline score is entered as a covariate [79]. We will also perform a per-protocol analysis as appropriate: In the NEMEX group, we define the per-protocol population as those participants who participated in at least 12 exercise sessions (that is, good compliance). In the Pharma group, we define the per-protocol population as those participants who used pharmacologic pain relief (acetaminophen or equivalent dose of NSAID) for more than 2,000 mg/daily on at least 28 days (that is, good compliance) during Pharma intervention. If fewer than 12 patients from each intervention group can be categorized as having good compliance, we will dichotomize the populations and compare the halves with the better compliance.

For binary response variables (for example, text-messaging question; drug use and adverse events), statistical significance will be tested by logistic regression analysis with treatment as the fixed-factor covariate.

### Data interpretation

To minimize bias, we have *a priori* decided how to interpret the possible variation in follow-up data scenarios: (a) If knee-joint load is reduced more in the NEMEX group compared with the Pharma group, then NEMEX is the preferred treatment; (b) if knee-joint load is reduced more in the Pharma group compared with the NEMEX group, then Pharma is the preferred treatment; or (c) if knee-joint load does not differ between the two treatment groups, the treatment associated with the greatest pain relief, functional improvement, and the fewest adverse events will be favored.

### Ethics and registration

This study is approved by the regional Committee for Medical Research Ethics, Project-ID: S-20110153 and the Danish Data Protecting Agency. The study is recorded at ClinicalTrials.gov, Identifier: NCT01638962. The Danish Medicines Agency has reviewed the protocol. The procedures followed are in accordance with the ethical standards of the responsible committee on human experimentation (institutional and national) and with the Declaration of Helsinki 1975, as revised in 2000. Because the intervention involves advice on optimal use of analgesics instead of a prescription of a specified dose, the study is considered a nonpharmacologic study and is therefore not required to undergo review by the Danish Medicines Agency’s external trial unit.

## Discussion

The need to develop efficacious treatment approaches for knee OA that are capable not only of relieving symptoms but also of slowing disease progression is an important research topic and clinical objective [80]. Our study builds on the premise that dynamic knee loading can be altered in individuals with mild to moderate knee OA. If knee loading can be reduced during functional weight-bearing ADLs, structural degeneration may be slowed in addition to achieving symptom relief. This study is, to our knowledge, the first RCT to compare the efficacy of two pain-relieving treatments with very different mechanisms of action, neuromuscular exercise and drug use, on knee load and function in people with mild to moderate knee OA.

Strengths of the study are the rigorous method, the pragmatic nature of the treatment delivery that takes place in several physiotherapy clinics and the self-administered use of pain-relieving drugs, and the reproducibility and feasibility of the exercise program [52]. The study is designed to have a high degree of external validity. The pragmatic recruiting procedure in which patients are recruited from GPs and advertisements in the local community is a strength because these people are often faced with a choice between these two treatment options.

A drawback of the pragmatic design is the inherent risk of recruiting a heterogeneous group, thereby leading to a negative result, even though heterogeneity is common in this clinical population.

It was recently shown that neuromuscular exercise does not reduce KAM in patients with moderate to severe knee OA and varus malalignment [30]. That study differs from the current one in two central aspects: primary outcome and patient group. In the study by Bennell *et al.* [30], the primary outcome was peak KAM (reflecting medial compartment load, using only the frontal plane), and the population had more-severe disease as reflected by KL grades 2 through 4 and varus malalignment. In the current study, the primary outcome is peak Knee Index (reflecting joint load across both compartments in all three planes), and the population had moderate to no radiographic knee OA (KL 0 to 3).

This study tests two different modalities of pain relief that might be important for the change in knee-joint load. Pharmacologic pain relief might increase joint loads [12, 13], and neuromuscular exercise might decrease knee-joint load.

The primary outcome of the Knee Index is a surrogate measure for the combined external load across both medial and lateral knee compartments. It should be noted that two participants can have the same absolute value on the Knee Index and have different medial compartment load, if, for instance, the relative contribution of the knee-adduction moment is different. The patient-reported outcome measures used in this study are chosen on the basis of their validity, feasibility, and relevance for the study aim [68–71, 74–77].

In the current study, the BMI cutoff of 32 or more was chosen for two reasons: to prevent difficulties of marker placement for the 3D-motion-capture analysis and to preclude the increased risk of soft-tissue movement artefacts [81]. Finally, weight loss before exercise maybe the more appropriate approach for the obese population.

To ensure a standardized recruitment procedure by all of the GPs and via advertisements, information meetings will be conducted before starting the recruiting process. The recruitment procedure is kept as simple as possible and consists of a standardized case-record form, which the GP is to complete and send to the project manager (BC). The recruitment through advertisements follows the same standardized case-report form that is completed and checked by a physician. The case-report form is designed to take no more than 5 minutes to complete.

To ensure adherence with the standardized exercise protocol from all participating physiotherapists, attendance at a 2-day course on OA knowledge and neuromuscular exercise is compulsory before study commencement. In addition, each physiotherapist will have conducted a few pilot exercise sessions together with the project manager (BC) to further ensure compliance with the exercise protocol across the participating physiotherapy clinics. During intervention and enrollment of participants, a weekly update email containing information on screened and recruited participants will be sent to all collaborators (GPs, physiotherapists, and chiropractors performing the radiographs). If recruitment slows, a dialogue with the relevant individual will be opened.

In addition, the study is adequately powered for the primary outcome measure, and our recruitment strategy will result in a sample typical of primary care. This trial, evaluating the possible modifying effects of a neuromuscular exercise program on joint load in people with mild to moderate knee OA, will improve our understanding of how neuromuscular exercise and advice on drug use affects knee-joint loads, self-reported outcomes, and functional performance.

## Trial status

The study is currently recruiting patients; about 45 participants have currently been enrolled and tested at baseline.

## Abbreviations

ADLs: Activities of Daily Living
BW: body weight
GPE: global perceived effect
HT: height
KL: Kellgren-Lawrence
KAM: knee adduction moment
NEMEX: NEuroMuscular EXercise
PASS: patient acceptable symptom state
Pharma: pharmacologic treatment
QOL: quality of life
NSAID: nonsteroidal antiinflammatory drug
OA: osteoarthritis
OUH: Odense University Hospital
SDU: University of Southern Denmark
VAS: visual analogue scale.
